# Inhibitory Effect on Cerebral Inflammatory Response following Traumatic Brain Injury in Rats: A Potential Neuroprotective Mechanism of N-Acetylcysteine

**DOI:** 10.1155/2008/716458

**Published:** 2008-05-06

**Authors:** Gang Chen, Jixin Shi, Zhigang Hu, Chunhua Hang

**Affiliations:** Department of Neurosurgery, Jinling Hospital, School of Medicine, Nanjing University, 305 East Zhongshan Road, Nanjing, Jiangsu 210002, China

## Abstract

Although N-acetylcysteine (NAC) has been shown to be neuroprotective for traumatic brain injury (TBI), the mechanisms for this beneficial effect are still poorly understood. Cerebral inflammation plays an important role in the pathogenesis of secondary brain injury after TBI. However, it has not been investigated whether NAC modulates TBI-induced cerebral inflammatory response. In this work, we investigated the effect of NAC administration on cortical expressions of nuclear factor kappa B (NF-*κ*B) and inflammatory proteins such as interleukin-1*β* (IL-1*β*), tumor necrosis factor-*α* (TNF-*α*), interleukin-6 (IL-6), and intercellular adhesion molecule-1 (ICAM-1) after TBI. As a result, we found that NF-*κ*B, proinflammatory cytokines, and ICAM-1 were increased in all injured animals. In animals given NAC post-TBI, NF-*κ*B, IL-1*β*, TNF-*α*, and ICAM-1 were decreased in comparison to vehicle-treated animals. Measures of IL-6 showed no change after NAC treatment. NAC administration reduced brain edema, BBB permeability, and apoptotic index in the injured brain. The results suggest that post-TBI NAC administration may attenuate inflammatory response in the injured rat brain, and this may be one mechanism by which NAC ameliorates secondary brain damage following TBI.

## 1. INTRODUCTION

Although traumatic brain injury (TBI) represents a significant public health problem in the world, there are currently no treatments that improve clinical outcome measures [1, 2]. Recently, several reports from experimental studies have demonstrated that N-acetylcysteine (NAC) played neuroprotective roles in TBI including restoration of TBI-induced mitochondrial dysfunction, increasing the reduced antioxidant enzyme, and protecting neurons [3–5]. Most of these previous studies focused on the modulation of NAC on oxidative stress in the brain following TBI, but ignored the influence of NAC on cerebral inflammation, which also played crucial roles in the mechanisms of secondary brain damage after TBI [6]. Furthermore, no study was found in the literature to investigate the effects of NAC administration on brain edema, blood-brain barrier (BBB) permeability, and cortical apoptosis following TBI.

Several clinical and experimental studies have demonstrated that secondary brain injury can be magnified by numerous immune mediators, which are frequently upregulated in response to TBI [7–9]. Increased levels of these molecules within the injured brain, including interleukin-1*β* (IL-1*β*), tumor necrosis factor-*α* (TNF-*α*), interleukin-6 (IL-6), and intercellular adhesion molecule-1 (ICAM-1), are believed to contribute to the cerebral damage, cell death, and BBB dysfunction [10]. A pivotal player in the regulation of these molecules is the nuclear factor kappa B (NF-*κ*B) family (cRel, RelA/p65, RelB, p50 and p52) of transcription factors [11, 12]. In our previous studies, we have demonstrated that cortical contusion trauma could induce a concomitant and persistent upregulation of NF-*κ*B, TNF-*α*, IL-6, and ICAM-1 in the injured rat brain, which might play a central role in the injury-induced inflammatory response of brain [13, 14].

NAC is a compound that increases the pool of glutathione. The latter is an important cellular antioxidant. It is a reactive oxygen species (ROS) scavenger and can restore the reduced cellular glutathione [15]. It is also cytoprotective since it inhibits the activation of NF-*κ*B and TNF-*α* production by lipopolysaccharide [16, 17]. Xiong et al. have reported that NAC could greatly restore brain glutathione levels and mitochondrial glutathione levels after TBI [3]. Also, in a rat model of experimental stroke, administration of NAC after ischemia onset protected the brain from free radical injury, apoptosis, and inflammation, with a wide treatment window [18]. Nevertheless, it is still unknown up till now whether NAC can affect the production of inflammatory agents in the brain after TBI.

The aim of the current study was to determine whether NAC could attenuate the TBI-induced brain inflammation in the pericontusional area. We hypothesized that the effect of NAC on modulating cerebral inflammatory response might be a mechanism by which NAC reduced cerebral edema, protected BBB, and repressed cortical apoptosis after TBI.

## 2. MATERIALS AND METHODS

### 2.1. Animals

Male Wistar rats (250–300 g) were purchased from Animal Center of Chinese Academy of Sciences, Shanghai, China. The rats were housed in temperature and humidity controlled animal quarters with a 12-hour light/dark cycle. All procedures were approved by the Institutional Animal Care Committee and were in accordance with the guidelines of the National Institutes of Health on the care and use of animals.

### 2.2. Experiment protocol

Rat model of cortical contusion trauma: following IP anesthesia with urethane (1000 mg/kg), animal head was fixed in the stereotactic frame. A right parietal craniotomy (diameter 5 mm) was drilled under aseptic conditions 1 mm posterior and 2 mm lateral to the bregma. We used a modification of Feeney's weight-drop model in which a freefalling weight onto the exposed intact cranial dura produced a standardized parietal contusion by letting a steel rod weighing 40 g with a flat end diameter of 4 mm fall onto a piston resting on the dura from a height of 25 cm [19]. The piston was allowed to compress the tissue a maximum of 5 mm. After operation procedures, the rats were then returned to their cages and the room temperature kept at 23 ± 1°C. Heart rate, arterial blood pressure, and rectal temperature were monitored, and the rectal temperature was kept at 37 ± 0.5°C, by using physical cooling (ice bag) when required, throughout experiments. Sham-operated rats were anesthetized and mounted in the stereotactic apparatus, with right parietal craniotomy surgically prepared alone and without brain injury.

**Figure 1:**
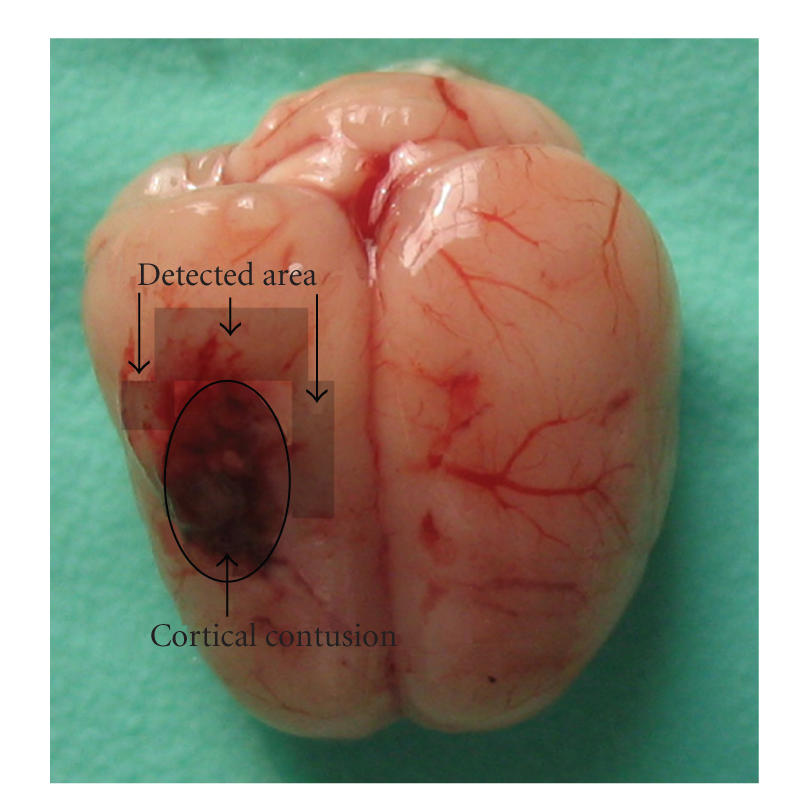
Schematic representation of the cortical contusion area induced by weight-dropping trauma and the studied region surrounding the injured brain.

We established 3 experimental groups in a randomized fashion: (a) the sham operation group (SHAM; *n* = 15), (b) the TBI group (TBI; *n* = 18), and (c) the NAC treatment group (TBI-NAC; *n* = 18). Rats of TBI-NAC group received injections of 150 mg/kg NAC IP at 15 minutes and 1, 2, and 3 days after the surgery. Rats of SHAM and TBI groups received equal volumes of 0.9% saline solution. The dose was chosen according to Hicdonmez et al. since they observed beneficial effects on preventing trauma-induced oxidative brain tissue damage following TBI after treatment with NAC using the same protocol [4]. The animals were decapitated 3 days after injury for tissue assays. The surrounding brain tissue of the injured cortex (see Figure 1) was dissected on ice as described in our previous study [14], some of which were put into 10% buffered formalin, the others were stored at liquid nitrogen immediately until use.

### 2.3. Nuclear protein extract and electrophoretic mobility shift assay (EMSA)

Nuclear protein was extracted and quantified as described [14, 20]. EMSA was performed using a commercial kit (Gel Shift Assay System; Promega, Madison, Wis, USA) following the methods in our laboratory. The NF-*κ*B oligonucleotide probe (5′-AGTTGAGGGGACTTTCCCAGGC-3′) was end-labeled with [*γ*-^32^P] ATP (Free Biotech, Beijing, China). EMSA was performed according to our previous study [14, 20].

### 2.4. Enzyme-linked immunosorbent assay (ELISA)

The levels of inflammatory mediators were quantified using specific ELISA kits for rats according to the manufacturers' instructions (TNF-*α* from Diaclone Research, Besançon, France; IL-1*β*, IL-6 from Biosource Europe SA, Nivelles, Belgium) and our previous studies [14, 20]. Values were expressed as ng/g protein.

### 2.5. Immunohistochemical study

Immunohistochemical studies were conducted on formalin-fixed, paraffin-embedded sections. The rabbit-antirat monoclonal antibody of ICAM-1 (diluted 1 : 100, Santa Cruz Biotechnology, Calif, USA) was used. Immunohistochemical assay was performed according to our previous study [14]. The positive cells were identified, counted, and analyzed under the light microscope by an investigator blinded to the grouping. The number of positive microvessels in each section was counted in 10 microscopic fields (at × 100 magnifications) and averaged for the positively immunostained vessel number of per visual field.

### 2.6. Brain water content

Brain edema was determined using the wet/dry method as previously described where the percentage of brain water = [(wet weight − dry weight)/wet weight] × 100% [21]. Briefly, brains were rapidly removed from the skull and the bilateral brains were separated. Both were placed separately into preweighed and labeled glass vials and weighed. The vials were then placed in an oven for 72 hours at 100°C and then reweighed to obtain dry weight content. The number of animals used in each group for brain edema study was as follows: for SHAM (*n* = 5), for TBI (*n* = 6), and for TBI-NAC (*n* = 6).

### 2.7. Blood-brain barrier permeability

Blood-brain barrier (BBB) permeability was determined by Evans blue (EB) extravasation at 3 days after TBI. Briefly, 2% Evans blue was injected IV at a dose of 2 mL/kg. Animals were then reanesthetized after 1 hour with urethane (1000 mg/kg) and perfused using saline to remove intravascular EB dye. Animals were then decapitated; the brains were removed and homogenized in phosphate buffered saline. Trichloroacetic acid was then added to precipitate protein, and the samples were cooled and centrifuged. The resulting supernatant was measured for absorbance of EB at 610 nm using a spectrophotometer. The number of animals used in each group for BBB permeability study was as follows: for SHAM (*n* = 5), for TBI (*n* = 6), and for TBI-NAC (*n* = 6).

### 2.8. TUNEL staining and quantitation of apoptotic cells

The formalin-fixed tissues were embedded in paraffin and sectioned at 4 *μ*m thickness with a microtome. The sections were detected for apoptotic cells by terminal deoxynucleotidyl transferase-mediated dUTP nick end labeling (TUNEL) method. TUNEL: in situ cell death detection kit POD (ISCDD, Boehringer Mannheim, Germany) was used. The procedures were according to the protocol of the kit and our previous study [14]. The positive cells were identified, counted, and analyzed under the light microscope by an investigator blinded to the grouping. The extent of brain damage was evaluated by apoptotic index which was the average number of TUNEL-positive cells in each section counted in 10 microscopic fields (at ×200 magnifications).

### 2.9. Statistical analysis

All data were presented as mean ± SD. SPSS 12.0 was used for statistical analysis of the data. All data were subjected to one-way ANOVA. Differences between experimental groups were determined by Fisher's LSD posttest. Statistical significance was inferred at *P* < .05.

## 3. RESULTS

### 3.1. EMSA for NF-*κ*B

**Figure 2:**
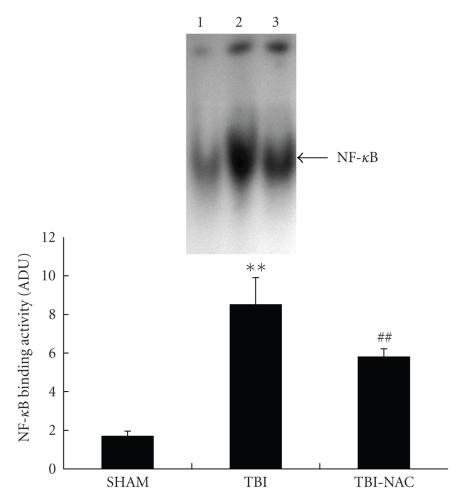
NF-*κ*B activity in the brain area surrounding the injury site in SHAM group (*n* = 5), TBI group (*n* = 6), and TBI-NAC group (*n* = 6). (Upper) EMSA autoradiography of NF-*κ*B DNA binding activity. (Bottom) levels of NF-*κ*B DNA binding activity quantified by computer-assisted densitometric scanning and expressed as arbitrary densitometric units (ADUs). As compared with SHAM group, NF-*κ*B binding activity measured by EMSA was significantly increased in TBI group. Compared to TBI group, NAC significantly suppressed NF-*κ*B activation in TBI-NAC group. **P < .01 versus SHAM group, ^##^ *P* < .01 versus TBI group.

EMSA autoradiography of NF-*κ*B DNA binding activity of the injured brain samples was shown in Figure 2. Low NF-*κ*B binding activity (weak EMSA autoradiography) was found in the SHAM group. Compared with SHAM group, NF-*κ*B binding activity in the injured brain was significantly increased (*P* < .01) in TBI group. In TBI-NAC group, the NF-*κ*B binding activity was significantly downregulated (*P* < .01) in the brain area surrounding the injury site after TBI.

### 3.2. Concentrations of IL-1*β*, TNF-*α*, and IL-6 in the injured brains

**Figure 3:**
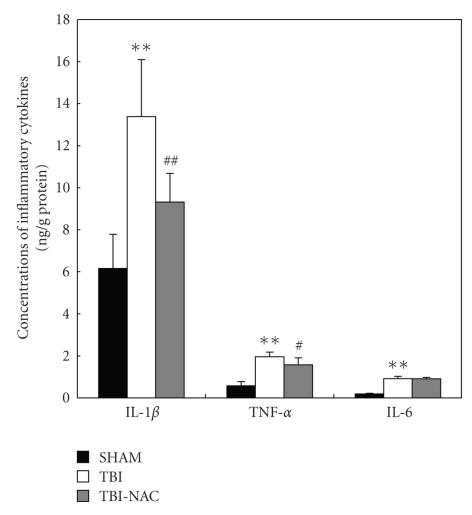
Changes of inflammatory mediators in the injured brains as determined by ELISA in SHAM group (*n* = 5), TBI group (*n* = 6), and TBI-NAC group (*n* = 6). TBI could induce the significantly increased concentrations of IL-1*β*, TNF-*α*, and IL-6 in the rat brain surrounding the injury site. In TBI-NAC group, the cortical concentrations of IL-1*β* and TNF-*α* but not IL-6 were markedly downregulated as compared with that of TBI group. **P < .01 versus SHAM group; ^#^ *P* < .05 and ^##^ *P* < .01 versus TBI group.

Concentrations of IL-1*β*, TNF-*α*, and IL-6 were low in the rat brains of SHAM group (6.16 ± 1.63, 0.58 ± 0.20, and 0.19 ± 0.03 ng/g protein, resp.) (see Figure 3). Compared with SHAM group, cortical levels of the three inflammatory cytokines were greatly induced after TBI (*P* < .01). As shown in Figure 3, NAC administration after TBI could lead to significantly decreased IL-1*β* and TNF-*α* concentrations, but had no significant effect on the IL-6 concentration (*P* > .05) in rat brain tissue.

### 3.3. ICAM-1 expression in the vessels of injured brain

**Figure 4:**
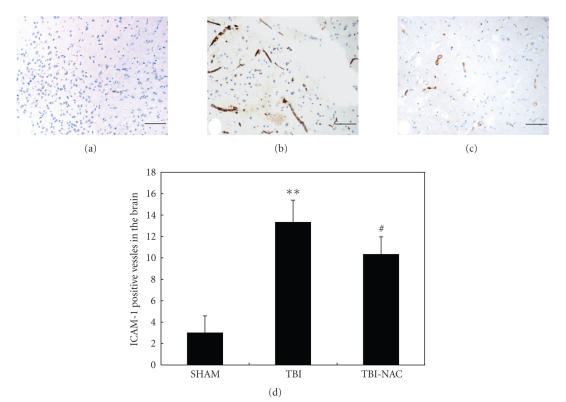
ICAM-1 immunohistochemistry in the injured cortex in SHAM group (*n* = 5), TBI group (*n* = 6), and TBI-NAC group (*n* = 6). (a) SHAM rats showing few ICAM-1 positive vessels. (b) TBI rats showing strong ICAM-1 positive vessels stained as brown. (c) TBI-NAC rats showing less ICAM-1 positive vessels than TBI rats (scar bar, 50 *μ*m). (d) Administration of NAC remarkably inhibited TBI-induced upregulation of ICAM-1 expression on cerebrovascular endothelia. **P < .01 versus SHAM group; ^#^ *P* < .05 versus TBI group.

As shown in Figure 4(a), few ICAM-1-immunostained cerebral microvessels were observed in the SHAM group rat brain. In TBI group, the number of ICAM-1-positive vessels was significantly increased as compared with that in the SHAM group (*P* < .01) (see Figures 4(b) and 4(d)). In TBI-NAC group, when compared with the TBI group, the number of ICAM-1-positive vessels was significantly decreased (*P* < .05) (see Figures 4(c) and 4(d)). The results showed that systemic injections of NAC could significantly downregulate the ICAM-1 immunoreactivity in the vessels of injured brain (see Figure 4(d)).

### 3.4. Brain water content

**Figure 5:**
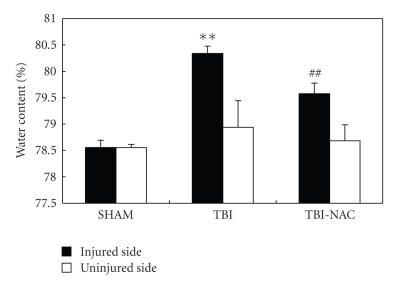
Alterations in brain water content in SHAM group (*n* = 5), TBI group (*n* = 6), and TBI-NAC group (*n* = 6). The brain water content of the injured side was increased significantly at 3 days after TBI. NAC treatment markedly reduced brain water content. No difference of brain water content was detected in the uninjured side among the three groups. **P < .01 versus SHAM group; ^##^ *P* < .01 versus TBI group.

Significant increase (*P* < .01) in water content was detected in the brain samples of injured side at 3 days after TBI when compared with sham-operated rats (see Figure 5). The mean value of brain water content in the injured side was decreased by NAC administration (*P* < .01) as compared with TBI group. For the uninjured side, the mean values of brain water content had no significant difference among SHAM, TBI, and TBI-NAC groups. The results suggested that post-TBI NAC treatment could attenuate brain edema in this rat TBI model.

### 3.5. Blood-brain barrier permeability

**Figure 6:**
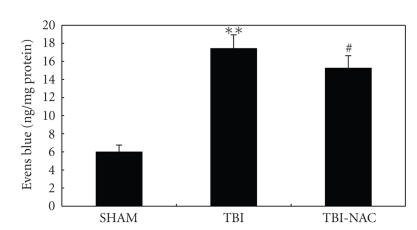
Alterations in Evans blue extravasation in SHAM group (*n* = 5), TBI group (*n* = 6), and TBI-NAC group (*n* = 6). TBI could induce a marked increase of BBB extravasation in the rat brain compared with SHAM group. After NAC administration, the Evans blue extravasation was significantly reduced as compared with TBI group. **P < .01 versus SHAM group, ^#^ *P* < .05 versus TBI group.

The pattern of Evans blue extravasation following TBI is shown in Figure 6. Rats in TBI group demonstrated a significant increase (*P* < .01) in BBB permeability to Evans blue relative to rats of SHAM group. Administration of NAC significantly inhibited Evans blue extravasation (*P* < .05), indicating a reduced BBB opening in response to NAC treatment.

### 3.6. Apoptosis in the injured cortex

**Figure 7:**
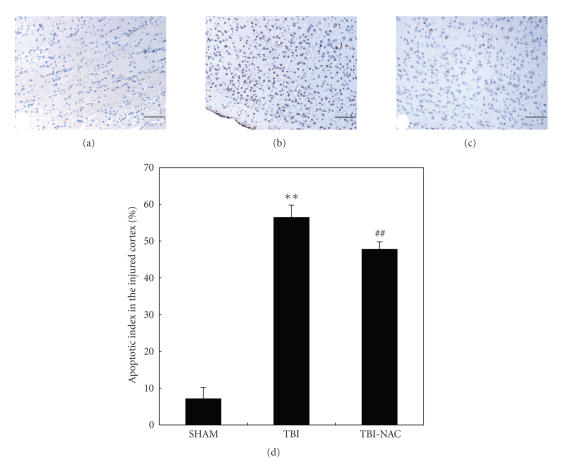
TUNEL immunohistochemistry staining of the injured cortex in SHAM group (*n* = 5), TBI group (*n* = 6), and TBI-NAC group (*n* = 6). (a) SHAM group rats showing few TUNEL apoptotic cells. (b) TBI group rats showing more TUNEL apoptotic cells stained as brown. (c) TBI-NAC group rats showing less TUNEL apoptotic cells than TBI group (scar bar, 50 *μ*m). (d) Administration of NAC significantly decreased the apoptotic index in rat injured brain following TBI. **P < .01 versus SHAM group; ^##^ *P* < .01 versus TBI group.

Few TUNEL-positive apoptotic cells were found in the SHAM group rat brain (see Figure 7(a)). In TBI group, the apoptotic index in the cortex surrounding the injured site was found to be significantly increased compared with that in SHAM animals (*P* < .01) (see Figures 7(b) and 7(d)). In TBI-NAC group, when compared with that in the TBI group, the apoptotic index in the studied cortex was significantly decreased (*P* < .01) (see Figures 7(c) and 7(d)). The result showed that NAC administration following TBI could lead less cell death in the brain surrounding the cortical contusion and was potential to ameliorate the secondary brain damage following TBI.

## 4. DISCUSSION

The main findings of this study are that (1) as common inflammation-related factors to TBI, NF-*κ*B, proinflammatory cytokines, and ICAM-1 were upregulated after TBI and could be suppressed when treated with NAC and (2) after NAC administration, the brain edema, BBB permeability, and apoptotic cell death were ameliorated. These findings suggest for the first time that NAC attenuates the TBI-induced cerebral inflammatory response and alleviates secondary brain damage following primary trauma in the rat TBI model.

There have been several studies focusing on the neuroprotective effects of NAC in TBI. As mentioned by Ellis et al. in their literature, NAC could direct scavenge radicals and stimulate glutathione peroxidase activity in a cat TBI model [22]. Their results suggested that NAC might be useful for treatment of oxygen-free radical-mediated brain injury. Another research indicated that NAC administered postinjury at an early stage could effectively restore TBI-induced mitochondrial dysfunction and the protective effect of NAC might be related to its restoration of glutathione levels in the brain [3]. More recent data revealed by Hicdonmez et al. was that NAC treatment after trauma was effective in lipid peroxidation, antioxidant enzyme activity, and neuronal protection in cerebral injury following closed head trauma [4]. In this current study, we found that NAC administration following TBI could reduce cerebral edema, BBB permeability, and apoptotic cell death which played important roles and were the major part in the secondary brain injury following TBI. However, despite the demonstrated mechanisms of NAC in neuroprotection, none of the previous studies focused on cerebral inflammatory response that might facilitate the development of secondary brain damage following primary trauma.

Brain inflammation represents only one of the numerous processes activated after TBI; it has not been fully elucidated due to the complexity of the large number of molecules shaping a sophisticated circuitry. The major effectors in this cascade are the proinflammatory cytokines that are usually released within minutes after challenge because they are stored intracellularly as precursor proteins eventually modified into active molecules. TBI damages the blood-brain barrier, then the blood cells such as neutrophils and macrophages accumulate in the brain and further sustain the cerebral inflammatory cascade [23]. Numerous immune mediators such as IL-1*β*, TNF-*α*, and IL-6 released within minutes of the primary injury can initiate the infiltration of inflammatory cells into the brain by activating ICAM-1 and other adhesion molecules [14]. The functional importance of NF-*κ*B in acute inflammation is based on its ability to regulate the promoters of a variety of genes whose products, such as IL-1*β*, TNF-*α*, IL-6, ICAM-1, and acute phase proteins, are critical to inflammatory processes [11]. NF-*κ*B activation enhances the transcription of proinflammatory cytokines, and the cytokines are known to in turn activate NF-*κ*B [24]. The positive feedback is believed to serve to amplify inflammatory signals. In our previous studies, it has been found that there were an early concomitant and persistent upregulation of NF-*κ*B binding activity, TNF-*α*, IL-6, and ICAM-1 expression in the injured brain after cortical contusion trauma [13, 14].

A number of studies have demonstrated that NAC could modulate inflammatory factors production such as NF-*κ*B, proinflammatory cytokines, and ICAM-1 [25]. Carroll et al. examined the influence of NAC on cerebral NF-*κ*B activity after temporary middle cerebral artery occlusion and indicated that activated NF-*κ*B was significantly increased 15 minutes after reperfusion in the affected hemisphere and could be abolished with NAC treatment [25]. Li et al. investigated effects of NAC on NF-*κ*B activation and proinflammatory cytokines production in protein malnutrition using an animal septic shock model and their results suggested that supplementation of NAC could normalize lipopolysaccharide-induced NF-*κ*B activation and proinflammatory cytokines production during early rehabilitation of protein malnourished mice [26]. Our study showed that cortical levels of NF-*κ*B, TNF-*α*, IL-1*β*, IL-6, and ICAM-1 were significantly induced by TBI at 3 days after trauma. Post-TBI NAC administration could repress the expressions of most of these inflammatory agents that accompanied TBI. Although our data have proved that NAC could downregulate the cerebral inflammatory factors after TBI, changes about the signaling pathways of inflammation and oxidative stress in the brain remain unknown. It is assured that further ingenious studies are needed and will be conducted in our laboratory.

Apoptosis in the traumatically injured brain occurs not only at the impact site but also as a result of secondary brain insults such as intracranial hypertension, hypoxia, or disturbances of microcirculation [27]. Studies regarding the effect of NAC on neural apoptosis after TBI have not been found to date. Nevertheless, several previous studies have demonstrated that NAC could prevent apoptotic death of neuronal cells in vitro [28, 29]. NAC also inhibited procaspase-9 processing and the activation of enzymatic activity of caspases induced by acrolein. Inhibition of acrolein-induced apoptosis using NAC was confirmed morphologically by diminished condensation of nuclear chromatin, as evaluated by fluorescence microscopy [30]. In this present study, we found that systemic NAC administration could inhibit apoptotic cell death in the injured brain and was potential to reduce the secondary brain damage following TBI. However, it is still unclear whether NAC can modulate the apoptotic signals and the whole mechanisms involved call for further research.

In conclusion, to the best of our knowledge, this is the first study to demonstrate the effect of NAC on the inflammatory response in the injured brain after TBI. We found that TBI could upregulate the expressions of NF-*κ*B, IL-1*β*, TNF-*α*, and ICAM-1 in the surrounding brain of injured site, which could be markedly inhibited by NAC administration. The therapeutic benefit of post-TBI NAC administration might be due to its salutary effect on modulating cerebral inflammation secondary to TBI.
